# Stemness and clinical performance of water-jet technology: A translational study in breast reconstruction

**DOI:** 10.1016/j.jpra.2025.10.010

**Published:** 2025-10-24

**Authors:** Francesco De Francesco, Nicola Zingaretti, Davide Quaglia, Giulio Moro, Annalaura Del Pin, Maria Pia Cavaleri, Andrea Giuseppe Ferraro, Giuseppe Andrea Ferraro, Emanuele Rampino Cordaro, Barbara Zavan, Michele Riccio, Pier Camillo Parodi

**Affiliations:** a Department of Reconstructive Surgery and Hand Surgery, Azienda Ospedaliera Universitaria delle Marche, Ancona, Italy; b Department of Medical Area (DAME), Clinic of Plastic and Reconstructive Surgery, Academic Hospital of Udine, University of Udine, Udine, Italy; c Department of Translational Medicine, University of Ferrara, Ferrara, Italy; d Plastic and Reconstructive Surgery Unit, Multidisciplinary Department of Medical-Surgical and Dental Specialties, University of Campania Luigi Vanvitelli, Naples, Italy; e Department of Medicine and Health Sciences “Vincenzo Tiberio”, Reconstructive and Aesthetic Plastic Surgery, University of Molise, Campobasso, Italy

**Keywords:** Adipose tissue, Autologous fat grafting, Water-jet-assisted liposuction, Body-jet, Fat processing, Liposuction, Lipotransfer, Mesenchymal stem cells, Translational research, Breast reconstruction

## Abstract

Autologous fat grafting has become integral to breast reconstruction, yet variability in graft retention continues to challenge its reliability. This study aimed to characterize the biological and clinical properties of adipose tissue harvested using Body-Jet water-assisted liposuction (WAL) technology. A cohort of 171 patients undergoing 206 lipofilling procedures was analyzed, alongside in vitro studies evaluating adipose-derived cell viability, proliferative capacity, and molecular profile. Lipoaspirates obtained using Body-Jet demonstrated high cellular viability, robust mesenchymal stem cell proliferation (doubling time ∼15 hours), and consistent trilineage differentiation (adipogenic, osteogenic, and chondrogenic). Gene expression and secretome analyses revealed a regenerative molecular signature, including balanced cytokine, growth factor, and extracellular matrix protein profiles. Clinically, the WAL approach was associated with low postoperative pain (mean NRS 3.17), minimal complications, and high patient satisfaction, as assessed using BREAST-Q outcomes. A significant correlation emerged between BMI and harvested fat volume, with no association between pain and BMI or donor site. These findings support the hypothesis that Body-Jet–based liposuction yields biologically viable and regenerative lipoaspirates, while offering favorable clinical tolerability. Although transient side effects such as edema and bruising were observed, the overall safety and quality of harvested tissue support the further use of WAL in fat grafting. Prospective controlled studies are needed to confirm long-term outcomes and comparative efficacy.

## Introduction

Breast reconstruction represents one of the most significant challenges in plastic and reconstructive surgery, with a central goal: restoring breast volume and symmetry while improving patients' quality of life. Among the available techniques, autologous fat grafting has emerged as a fundamental tool due to its ability to integrate tissue volumes in a natural and safe manner. However, variability in the survival of transplanted fat and the need to enhance clinical outcomes have driven research toward optimizing methods for harvesting and processing adipose tissue.^1^, ^2^, ^3^, ^4^, ^5^

The Body-jet technology, based on water-jet-assisted liposuction, has opened new horizons in the field of fat grafting. This technique utilizes a pulsating low-pressure water jet to gently detach adipose cells from connective tissue, minimizing mechanical trauma. The result is adipose tissue characterized by high cell viability, a critical factor for the survival of fat grafts post-transplantation.^6^ Comparative studies have demonstrated that water-jet-assisted liposuction better preserves the stromal vascular fraction (SVF) compared to conventional techniques. The SVF contains a significant concentration of mesenchymal stem cells derived from adipose tissue (adipose-derived stem cells, ASCs), known for their regenerative, angiogenic, and anti-inflammatory properties.^3^^,^^7^

Adipose-derived stem cells play a key role in tissue regeneration due to their ability to differentiate into adipocytes, promote angiogenesis, and support fat graft survival. These properties are particularly relevant in breast reconstruction, where fat grafting is often used not only to restore breast volume but also to improve tissue quality compromised by surgery or radiotherapy.^8^, ^9^, ^10^, ^11^, ^12^, ^13^ The use of Body-jet technology, which enhances the quality of harvested adipose tissue, therefore represents a potential breakthrough in breast reconstruction, offering greater predictability in outcomes and reducing the need for repeated interventions.^14^

Breast reconstruction through fat grafting requires careful evaluation of the harvested adipose tissue and its ability to regenerate surrounding tissues. The Body-jet technology stands out not only for improving adipose cell viability but also for reducing the incidence of intra- and postoperative complications. The low volume of infiltrated fluids during the procedure and reduced mechanical stress make this technique less invasive, while simultaneously improving the quality of the harvested fat.^15^ Moreover, the Body-jet’s ability to preserve a high number of CD34+ cells (markers of stem and progenitor cells) highlights its regenerative potential.^7^

Preclinical and clinical studies have shown that adipose tissue harvested with the Body-jet promotes better angiogenesis and increases fat graft survival, partly attributable to the preservation of the stromal vascular fraction. In vivo, these effects translate into improved integration of the transplanted fat tissue and enhanced local microvascular conditions, both of which are essential for the success of breast reconstruction.^6^^,^^16^^,^^17^

This study aims to explore the application of Body-jet technology in breast reconstruction, analyzing its impact on the quality and viability of transplanted adipose tissue, as well as on long-term clinical outcomes. Through an approach that integrates technological innovation and regenerative medicine, we hope to contribute to improving the standards of care in breast reconstructive surgery. The combination of gentle adipose harvesting with the extraordinary regenerative capabilities of adipose-derived stem cells could represent a breakthrough in managing patients undergoing breast reconstruction, reducing the risk of fat resorption, and ensuring optimal aesthetic and functional results. Moreover, we report our own experience to validate that reported in the literature.

## Materials And methods

### Study design

This study was structured in two parts: (1) a retrospective clinical analysis of patients who underwent fat grafting procedures with the Body-Jet system between January 2015 and February 2025; and (2) a prospective laboratory investigation assessing the biological characteristics of adipose tissue harvested with the same system. For the in vitro analysis, adipose tissue samples were obtained from a subset of consenting patients under an ethically approved research protocol (Approval Code: 132903, University of Ferrara). All participants included in the in vitro component provided written informed consent for the collection and use of their tissue samples.

### In vitro analysis

#### Harvesting of fat tissue

**Figure 1:**
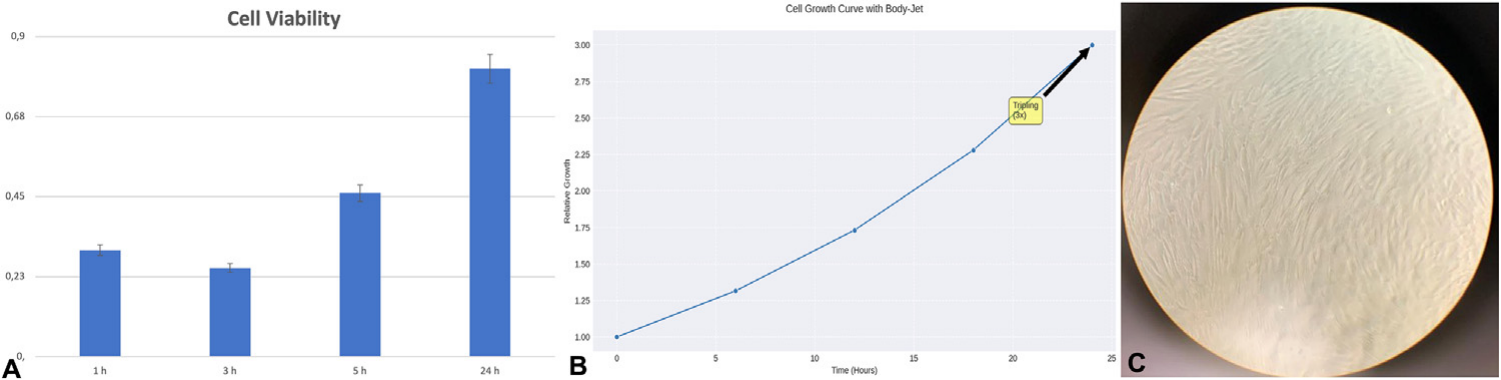
Cell culture of lipoaspirate with Body Jet shows: A. A high proliferation index with the MTT test, tripling in 24 hrs; B. Cell Growth. The average growth rate is 0.0833 units per hour with a doubling time of 15.14 hours; C. Cell culture expansion with high concentration of fibroblast like cells typical of adipose stem cells.

Lipoaspirates for the in vitro analysis were collected from a subset of healthy female patients undergoing water-jet–assisted liposuction for reconstructive purposes. All participants provided written informed consent, and the study received approval from the local Ethics Committee. Lipoaspirates were collected exclusively using water-jet–assisted liposuction (Body-Jet system, human med AG, Schwerin, Germany) for all patients enrolled in the in vitro component of the study. The procedure was performed on a standardized anatomical area using the LipoCollector device (Fig. 1), ensuring gentle harvesting and preservation of cell viability. The lipoaspirates were extracted by a single surgeon using identical equipment and materials, with the only variable being the use of water-jet assistance. Initially, a 2-mm cannula was employed for tumescent infiltration (a solution containing 1000 ml of saline, 1 ml of 1:200,000 adrenaline, and 600 mg of lidocaine) into the subcutaneous fat of each donor's side at jet speed level 2 (level 1 being the lowest). Subsequently, a 3.8-mm cannula with suction openings of 0.9 mm was used to aspirate fat particles under a negative pressure of −0.5 bar (1 bar = 100 kPa). All samples were processed in a blinded manner, with coded labeling to maintain operator objectivity.

### Isolation and expansion of cells

The lipoaspirate was processed for enzymatic digestion and cell isolation following a standardized procedure. Initially, the adipose tissue was washed with phosphate-buffered saline (PBS, EuroClone, Milan, Italy). Enzymatic digestion was performed using a 0.075 % collagenase type II solution derived from *Clostridium histolyticum* (Sigma-Aldrich, St. Louis, MO, USA) diluted in Hank's balanced salt solution (HBSS, Lonza S.r.l., Milan, Italy). The digestion was carried out at room temperature under gentle agitation for 3 hours. The collagenase activity was neutralized by adding an equal volume of complete DMEM (cDMEM), which consisted of Dulbecco’s Modified Eagle’s Medium (DMEM, Lonza S.r.l.) supplemented with 10 % fetal bovine serum (FBS, Beached S.p.A., Milan, Italy) and 1 % penicillin/streptomycin (P/S, EuroClone). The sample was centrifuged at 1200 rpm for 4 min, and the resulting pellet was washed with PBS and filtered through a 70 μm cell strainer (BD Biosciences, Mississauga, Ontario, Canada). The cell suspension was resuspended in cDMEM, transferred to a 25 cm² tissue culture flask, and incubated at 37 °C with 5 % CO2. After 3 days, non-adherent cells were removed, and fresh medium was added to the adherent cells. Upon reaching confluence, adipose-derived stem cells (ADSCs) were harvested using trypsin treatment and cultured up to passage 3.

### Cell culture and proliferation assay

Cell proliferation was evaluated using a modified version of the MTT (methyl thiazolyl-tetrazolium) assay, following an adapted protocol from Denizot and Lang (Denizot F and Lang R, J Immunol Methods 1986). This assay measures mitochondrial activity, as only functional mitochondria can convert the MTT solution into a characteristic blue-violet product. Following the removal of culture medium, cells were exposed to 1 mL of MTT solution (0.5 mg/mL in PBS) and incubated for 3 hours at 37 °C. After the incubation period, the MTT solution was carefully removed, and the samples were treated with 0.5 mL of a solution containing 10 % dimethyl sulfoxide in isopropanol for 30 min at 37 °C. The absorbance measurements were performed using a multilabel plate reader (Victor 3, Perkin Elmer, Waltham, MA, USA). For each sample, 200 μL aliquots were transferred to 96-well plates, and absorbance readings at 570 nm were recorded in duplicate. The assessment was conducted at multiple time points: 7, 14, 21, and 28 days of culture. Cell growth was quantified, and parameters such as doubling time, growth rate, and coefficient of determination (R²) were calculated to characterize the proliferative capacity of the cell population.

### Immunophenotyping

For the analysis of surface antigens, total RNA was isolated from each sample using the TRIzol® Reagent (Invitrogen, Carlsbad, CA, USA) and quantified with a NanoDrop spectrophotometer (NanoDrop™ 1000, Thermo Scientific, Waltham, MA, USA). For first-strand cDNA synthesis, 500 ng of total RNA was reverse transcribed using M-MLV Reverse Transcriptase (Moloney Murine Leukemia Virus, Invitrogen, Paisley, UK) following the manufacturer’s instructions.

Primers for the target genes were designed using Primer 3 software, and the sequences used are as follows:

**Table:** 

Gene	Forward primer (5′-3′)	Reverse primer (5′-3′)
FGF2	AGAAGAGCGACCCTCACATCA	CGGTTAGCACACACTCCTTTG
INS	GCAGCCTTTGTGAACCAACAC	CCCCGCACACTAGGTAGAGA
LIF	TGCCAATGCCCTCTTTATTC	GTCCAGGTTGTTGGGGAAC
POU5F1	GTGGAGAGCAACTCCGATG	TGCAGAGCTTTGATGTCCTG
SOX2	GCCGAGTGGAAACTTTTGTCG	GGCAGCGTGTACTTATCCTTCT
TERT	CGGAAGAGTGTCTGGAGCAA	GGATGAAGCGGAGTCTGGA
WNT3A	CTCGCTGGCTACCCAATTTG	CTTCACACCTTCTGCTACGCT
ALCAM	ACGATGAGGCAGACGAGATAAGT	CAGCAAGGAGGAGACCAACAA
ANPEP	GCTGCTGCTGCTGCTGCT	GCTGCTGCTGCTGCTGCT
BMP2	AAGCCGAGCCGCAGCTTCTT	GCTGCTGCTGCTGCTGCT
CASP3	GCTGCTGCTGCTGCTGCT	GCTGCTGCTGCTGCTGCT
ENG	GCTGCTGCTGCTGCTGCT	GCTGCTGCTGCTGCTGCT
ERBB2	GCTGCTGCTGCTGCTGCT	GCTGCTGCTGCTGCTGCT
FUT4	GCTGCTGCTGCTGCTGCT	GCTGCTGCTGCTGCTGCT
ITGA6	CCAGTGGTGTCTGTCGCTAA	CTGAGTCTGAGTGCCGTGAT
ITGAV	GCTGCTGCTGCTGCTGCT	GCTGCTGCTGCTGCTGCT
KDR	GCTGCTGCTGCTGCTGCT	GCTGCTGCTGCTGCTGCT
MCAM	GGGTACCCCATTCCTCAAGT	CAGTCTGGGACGACTGAATG
NGFR	GCTGCTGCTGCTGCTGCT	GCTGCTGCTGCTGCTGCT
NT5E	GCTGCTGCTGCTGCTGCT	GCTGCTGCTGCTGCTGCT
PROM1	GCTGCTGCTGCTGCTGCT	GCTGCTGCTGCTGCTGCT
THY1	ATGAAGGTCCTCTACTTATCCGC	GCACTGTGACGTTCTGGGA
VCAM1	GGGAAGATGGTCGTGATCCTT	TCTGGGGTGGTCTCGATTTTA
GAPDH	GAAGGTGAAGGTCGGAGTCA	GAAGATGGTGATGGGATTTC

Real-time PCR was performed with the designed primers at a concentration of 300 nM using FastStart SYBR Green Master (Roche Diagnostics, Mannheim, Germany) on a Rotor-Gene 3000 (Corbett Research, Sydney, Australia). The thermal cycling conditions were: an initial denaturation at 95 °C for 15 min, followed by 40 cycles of denaturation at 95 °C for 15 s, annealing at 60 °C for 30 s, and elongation at 72 °C for 20 s. Gene expression levels were normalized to the internal reference gene GAPDH, whose expression remained stable under the experimental conditions.

The following markers were evaluated for evaluating the stemness properties: FGF2, INS, LIF, POU5F1, SOX2, TERT, and WNT3A. Additionally, the expression of mesenchymal stem cell (MSC)-specific markers was analyzed across 16 key proteins: ALCAM, ANPEP, BMP2, CASP3, ENG (CD105), ERBB2, FUT4, ITGA6, ITGAV, KDR, MCAM, NGFR, NT5E (CD73), PROM1, THY1 (CD90), and VCAM1. The percentage of positive cells for each marker was quantified, and the data were presented as mean ± standard deviation (SD). Statistical analysis was performed using the Kruskal-Wallis test to determine any significant differences between the marker expression levels and one-way ANOVA and Tukey's post-hoc test to identify significant differences between the groups.

All experiments were conducted using three independent cell preparations and were repeated at least three times.

### Differentiation assays

Expanded cells from passage 3 were utilized for differentiation studies. Each differentiation protocol was performed in triplicate at designated time points.

For *adipogenic differentiation*, cells were plated at a density of 5000 cells per well in 12-well plates containing slides for adherent growth. Following 24-h incubation (37 °C, 5 % CO_2_), standard media was replaced with adipogenic induction media (Sigma-Aldrich).

*Chondrogenic differentiation* was initiated by seeding 1 × 106 cells in 5 µl of complete media per well. After initial attachment, StemPro chondrogenic media (GIBCO) was added. Media changes occurred every third day.

For *osteogenic induction*, wells were seeded with 5000 cells in standard media. After 24 hours, media was changed to StemPro osteogenic media (GIBCO).

RNA from adipogenic, chondrogenic and osteogenic differentiated cells was isolated, reverse transcribed, and analyzed for mRNA expression of PPARG, zfp467, and RHOA for adipogenic differentiation, BMP2, GDF5, GDF6, GDF7, ITGAX, ABCB1, BMP4, and KAT2B for chondrogenic differentiation, BMP2, KDR, FGF10, RUNX2, SMURF1, SMURF2, and PTK2 per osteogenic differentiation and GAPDH using a real-time polymerase chain reaction system. All experiments were performed in triplicate. Primers for the target genes were designed using Primer 3 software, and the sequences used are as follows:

**Table:** 

Gene	Forward primer (5′-3′)	Reverse primer (5′-3′)
PPARG	GGGATCAGCTCCGTGGATCT	TGCACTTTGGTACTCTTGAAGTT
ZFP467	CCACTGTGACATCTGCAAGG	CCAGGTGGTCTTCCTCTTCA
RHOA	GGAAAGCAGGTAGAGTTGGCT	GGCTGTCGATGGAAAAACACAT
BMP2	AAGCCGAGCCGCAGCTTCTT	GCTGCTGCTGCTGCTGCT
GDF5	CAGCGTGAAGCGCTATCTC	ACTCGTGCCTCTGGTCATC
GDF6	AGCAGGACGAGGCATCTAAG	TCACAGTAGTTGGCAGCGTC
GDF7	CTGAAGCCTCAGAGTCAGCA	GTTGTAGCGGCAGTTGAGTG
ITGAX	GTTCTGGAGCCACACACTGT	GCTGCCCACGGTAAAGATGA
ABCB1	TTGCTGCTTACATTCAGGTTTCA	AGCCTATCTCCTGTCGCATTA
BMP4	TGGTCTTGAGTGCCTGCAGT	CTGAGGTTAAAGAGGAAACGA
KAT2B	CTGGACGTGGAATGTGCTG	CATGTTTCGGAGCTGTGAGG
KDR	GGCCCAATAATCAGAGTGGCA	CCAGTGTCATTTCCGATCACTTT
FGF10	ATGTCCGCTGGAGAAAGCTA	CCCCTTCTTGTTCATGGCTA
RUNX2	TGGTTACTGTCATGGCGGGTA	TCTCAGATCGTTGAACCTTGCTA
SMURF1	CGAGGTTCTGGTCAGCATTT	GGTACCAGATCCTGCTGTCC
SMURF2	GTCCAGAGACCGAACACCAT	GTGCGTGTCGTGGTAGTTCT
PTK2	GTCTGCCTTCGCTTCACG	GAATTTGTGTTGGCTCTGTCG
GAPDH	GAAGGTGAAGGTCGGAGTCA	GAAGATGGTGATGGGATTTC

### Secretome analysis

Lipoaspirate samples obtained using Body-jet technology were processed following the device manufacturer's protocol. After processing, the samples were concentrated via centrifugation, and the resulting pellet was used for comprehensive protein analysis. Sample supernatants were collected and centrifuged (400 × g, 10 min, 4 °C) prior to freezing for subsequent analysis. Multiple protein categories were evaluated using commercial ELISA kits (Thermo Fisher Scientific, Waltham, MA, USA) according to manufacturer's protocols: Cytokines & Chemokines: CXCL2, CXCL5, IL-1β, IL-2, IL-4, IL-6, IL-10; Growth Factors: CSF2, CSF3, CTGF, FGF2, FGF7, FGF10, HBEGF, HGF, IGF1, MIF, TGFA, TGFβ1, TNF; ECM Components: COL1A1, COL1A2, COL3A1, COL4A1, COL4A3, COL5A1, COL5A2, COL14A1, VTN; Remodeling Enzymes: MMP1, MMP7, PLAT

Optical density measurements were performed at 405 nm or 450 nm using a Victor 3 plate reader.

### Statistical analysis

All statistical analyses were performed using R (version 4.0.2) and GraphPad Prism (version 9.0). Data were expressed as mean ± standard deviation (SD) unless otherwise specified, and statistical significance was set at *p* < 0.05. Cell growth analysis was conducted using non-linear regression with an exponential growth model, from which population doubling time and growth rate were calculated. The goodness of fit was assessed using the coefficient of determination (R²). For marker expression analysis, we employed multiple statistical approaches. The Kruskal-Wallis test was used to compare stemness marker expression across groups, while one-way ANOVA followed by Tukey's post-hoc test was applied for MSC-specific marker analysis. Pearson correlation coefficients were calculated to evaluate relationships between different markers. Moreover, A possible correlation, using Pearson’s correlation was investigated between patient BMI and the amount of fat harvested, BMI and pain reported 24 hours after the procedure, harvesting site and the amount of fat obtained for infiltration, harvesting site and pain reported 24 hours after the procedure and finally amount of fat obtained for infiltration and pain reported 24 hours after the procedure.

Gene expression during differentiation was analyzed using one-way ANOVA, with Tukey's post-hoc test for multiple comparisons between groups. For adipogenic (PPARG, zfp467, RHOA), osteogenic, and chondrogenic gene expression analyses, statistical significance was established at *p* < 0.05. ELISA data were analyzed using one-way ANOVA with Bonferroni correction for multiple comparisons. Data distribution was assessed using the Shapiro-Wilk normality test. Sample size was determined through power analysis using G*Power (version 3.1), with assumptions of α = 0.05 (type I error), β = 0.20 (power = 0.80), and medium effect size (d = 0.5). Data visualization was accomplished using ggplot2 in R for gene expression analyses, GraphPad Prism for box plots and correlation analyses.

All experiments were performed in triplicate, with outliers identified using Grubbs' test (α = 0.05). Reproducibility was assessed through calculation of inter- and intra-assay coefficients of variation (CV).

### Clinical evaluation and patient-reported outcomes

The clinical evaluation was conducted on a cohort of 171 female patients who underwent lipofilling with the Body-Jet system during breast reconstruction at our hospitals between January 2015 and February 2025. Only patients treated exclusively with the Body-Jet lipofilling technique, without concurrent surgical procedures, were included in the analysis.

Patient data were retrieved from electronic medical records, examination reports, and paper documentation. A dedicated database was compiled to systematically record anthropometric and clinical characteristics, primary surgical interventions, fat harvesting sites, harvested fat volume, postoperative complications, and pain scores assessed via the Numeric Rating Scale (NRS).

Anthropometric parameters included age and Body Mass Index (BMI, kg/m²), with metabolic status categorized as normal weight (BMI 18.5–24.9), overweight (BMI 25–30), and obese (BMI ≥30). The study also considered adjuvant therapies, including chemotherapy, radiotherapy, and anti-hormonal therapy, as well as the occurrence of disease recurrence.

Surgical data encompassed the type of breast resection (e.g., skin-sparing, NAC-sparing, skin-reducing mastectomy, simple mastectomy, quadrantectomy), reconstruction method (autologous flap-based or prosthetic), and indications for lipofilling (e.g., volume deficits, capsular contracture, wrinkling/rippling, pain, tissue retraction, vascular compromise). The fat harvesting site and volume were documented, along with postoperative pain at 24 hours, assessed via the NRS scale.

Postoperative complications were evaluated through follow-up examinations, focusing on early complications at both donor and recipient sites. Patient satisfaction was assessed using the BREAST-Q 2.0 questionnaire, administered electronically via Google Forms following prior telephone contact.

## Results

### In vitro analysis

#### Cell viability and cell growth

The Figure 1 presents an analysis of the cellular proliferation of lipoaspirate processed with Body Jet technology. The Figure focuses on two main aspects: i) Evaluation of the kinetics of cellular proliferation through MTT assay (Figure 1A,B); ii) Morphological characterization of cell populations in culture (Figure 1C).

The visualization shows the 24-h cell growth curve, which presents an exponential trend characteristic of cell proliferation (Figure 1A,B). The study highlighted a significant proliferative capacity of cells derived from lipoaspirate processed with Body-Jet technology, revealing an exponential growth pattern, a tripling of the cell population in 24 hours with a doubling time of approximately 15 hours and a constant growth rate of 0.083 units/h. These parameters are indicative of a highly vital and proliferative cell population, a characteristic typically associated with adipose-derived mesenchymal stem cells. The rapid growth kinetics suggests optimal preservation of cell viability during the lipoaspiration process, an expression of a high quality of the biological sample and a significant potential. Moreover, the dataset presents an analysis of lipoaspirate cell culture using Body Jet technology. The data focuses on cell proliferation. In particular, the proliferative capacity is highlighted through the MTT test and the presence of fibroblast-like cells (Figure 1B). The analysis of cell growth revealed a highly predictable and reproducible exponential growth model, with a coefficient of determination (R²) of 1.0000, indicating a perfect fit to the mathematical model. The initial cell population (N0) was estimated at 1.0000 units, with an exponential growth rate (r) of 0.0458 per hour. This corresponds to a cell population doubling time of approximately 15.14 hours.

The analysis of growth rate dynamics (Supplemental Figure 2A,B) showed an average instantaneous growth rate of 0.0833 units/h, with a relatively low standard deviation (0.0252 units/h), indicating minimal variability in the cell proliferation process. The growth rate ranged between 0.0527 and 0.1201 units/h, with the maximum observed during the intermediate phase of the culture period, suggesting optimal cell vitality during this phase.

### Immunophenotype

**Figure 2:**
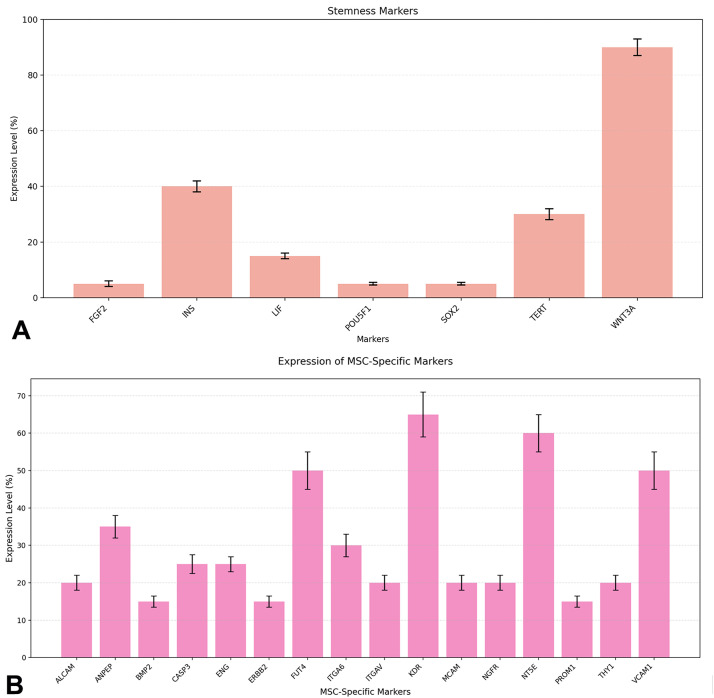
A. Expression analysis of stemness markers. Expression levels of different stemness markers were analyzed and quantified as percentage ( %). Data are presented as mean ± standard deviation (SD). The markers analyzed include FGF2, INS, LIF, POU5F1, SOX2, TERT, and WNT3A. WNT3A showed the highest expression (90 % ± 3 %), followed by medium expression markers INS (40 % ± 2 %) and TERT (30 % ± 2 %), while FGF2, POU5F1, and SOX2 exhibited the lowest expression levels (5 % ± 0.5–1 %). Statistical analysis was performed using Kruskal-Wallis test (H = 6.000, *p* = 0.4232); B. Expression levels of MSC-specific markers across 16 key proteins, categorized into high, moderate, and low expression groups. The graph highlights significant variability in marker expression, with KDR and NT5E showing the highest levels, suggesting their prominent role in MSC functionality.

The analysis of stemness markers expression (Figure 2A) revealed a heterogeneous pattern of expression, with levels ranging from a minimum of 5 % to a maximum of 90 %. The marker WNT3A showed the highest level of expression (90 %), while FGF2, POU5F1, SOX2 showed the lowest levels of expression (5 %). The overall mean of expression was 27.1 % (±30.9 % SD).

The Kruskal-Wallis test indicated no statistically significant differences between the groups (H = 6.000, *p* = 0.4232, α = 0.05). This lack of significance may be attributed to the limited sample size and high variability among markers. The data suggest that the extracted and enzymatically digested adipose cells possess a residual regenerative capacity, as evidenced by the expression of TERT and the predominance of WNT3A. However, the low levels of POU5F1 and SOX2 indicate that these cells are predominantly differentiated, with a limited presence of pluripotent stemness. The high level of INS may reflect the metabolic nature of the adipose cells. This profile supports the idea that adipose cells contain a subpopulation of cells with stemness potential, influenced by their environment and by the treatment undergone with the bodyjet system. The hierarchical clustering analysis of stemness markers (Supplemental Figure 3A) reveals a clear stratification into three distinct groups based on their expression levels. WNT3A stands out as the sole member of the high-expression cluster, with a significantly elevated expression level of 90 %, underscoring its potential pivotal role in maintaining stemness properties. The medium-expression cluster includes INS (40 %) and TERT (30 %), which may represent secondary regulators or co-factors in stemness pathways. Lastly, the low-expression cluster comprises LIF (15 %), FGF2, POU5F1, and SOX2 (all at 5 %), suggesting these markers may play supportive or context-dependent roles in stemness regulation. The use of Euclidean distance and Ward linkage methods highlights the hierarchical relationships among these markers, emphasizing the dominant role of WNT3A and the relative contributions of other markers in the stemness network.

Moreover, the analysis of MSC-specific markers expression revealed a distinctive profile across multiple stemness-related proteins (Figure 2B). The analysis encompassed sixteen key markers: ALCAM, ANPEP, BMP2, CASP3, ENG (CD105), ERBB2, FUT4, ITGA6, ITGAV, KDR, MCAM, NGFR, NT5E (CD73), PROM1, THY1 (CD90), and VCAM1. The expression pattern showed notable variability, with values ranging from 15 % to 65 %. Among these, KDR demonstrated the highest expression (65 %), followed by NT5E (60 %) and FUT4 (50 %), with standard deviations ranging from 1.5 to 6 %. Conversely, markers such as BMP2 and ERBB2 exhibited lower expression levels (approximately 15 %). The expression profile can be stratified into three main groups (ANOVA, F=54.4, *p* < 0.001): High expression markers (>50 %): KDR (65 %), NT5E (60 %), FUT4 (50 %), and VCAM1 (50 %) (Tukey HSD, *p* < 0.001); Moderate expression markers (25–35 %): ANPEP (35 %), ITGA6 (30 %), CASP3 (25 %), and ENG (25 %) (Tukey HSD, *p* < 0.05); Low expression markers (<20 %): BMP2, ERBB2, PROM1 (all approximately 15 %–20 %). Statistical analysis revealed significant variations in expression levels among different markers (*p* < 0.05). The presence of these markers, particularly the high expression of KDR and NT5E, suggests robust stemness characteristics and potential angiogenic properties. The moderate expression of adhesion molecules like ANPEP and ITGA6 indicates preserved cell-cell and cell-matrix interaction capabilities. Tukey's post-hoc analysis (Supplemental Figure 3B) confirmed statistically significant differences among all groups, with a mean difference of 38.75 % between high and low expression groups (*p* < 0.001), 27.5 % between high and moderate groups (*p* < 0.001), and 11.25 % between moderate and low groups (*p* < 0.05). This hierarchical stratification in marker expression suggests a differential regulation of the various molecular pathways involved in maintaining mesenchymal stem cell characteristics.

### Differentiation

#### Adipogenic differentiation

**Figure 3:**
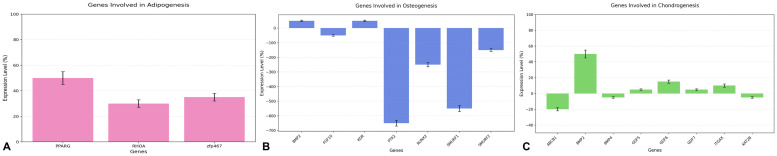
A. Expression levels of genes involved in adipogenesis (PPARG, zfp467, and RHOA) with error bars indicating variability. PPARG shows the highest expression, followed by zfp467 and RHOA, reflecting their roles in adipogenic differentiation; B. Expression analysis of genes involved in osteogenesis showing differential regulation of key factors. BMP2 exhibits positive expression, while other genes show varying degrees of negative regulation, reflecting the complex molecular control of osteogenic differentiation (ANOVA, *p* < 0.01); C. Expression analysis of genes involved in chondrogenesis showing differential regulation of key factors. BMP2 exhibits the highest positive expression, followed by moderate expression of GDF family members and ITGAX, while ABCB1 shows notable negative regulation, reflecting the molecular control of chondrogenic differentiation (ANOVA, *p* = 0.0629).

The analysis of gene expression involved in adipogenesis revealed a hierarchical pattern among the three genes analyzed: PPARG, zfp467, and RHOA (Fig. 3A). PPARG exhibited the highest expression level (50 %, SD ±5 %), significantly greater than both zfp467 (35 %, SD ±3 %) and RHOA (30 %, SD ±3 %) as confirmed by Tukey's post-hoc test (*p* < 0.001). zfp467, with an intermediate expression level, was also significantly higher than RHOA (*p* < 0.001). The ANOVA results (F-statistic = ∞, *p* < 0.001) confirmed statistically significant differences among the three groups. The hierarchical expression pattern (PPARG > zfp467 > RHOA) aligns with the sequential activation of molecular pathways during adipogenesis. The significant differences in expression levels (*p* < 0.001) highlight the distinct and complementary roles of these genes in orchestrating the differentiation process. This pattern underscores a well-coordinated adipogenic program, with PPARG driving the process, zfp467 supporting transcriptional regulation, and RHOA facilitating structural adaptation.

#### Osteogenic differentiation

The analysis of gene expression involved in osteogenesis revealed a complex pattern among seven key genes. The one-way ANOVA demonstrated significant differences among the expression groups (F = 14.6275, *p* = 0.0049). BMP2 showed the highest positive expression (50 % ± 5 %), significantly different from the other genes. The remaining genes showed varying degrees of negative expression, with KDR showing slight negative expression (-20 % ± 5 %), followed by FGF10 (-50 % ± 5 %), and SMURF2 (-150 % ± 10 %). More pronounced negative expression was observed in RUNX2 (-250 % ± 15 %), SMURF1 (-550 % ± 20 %), and PTK2 (-650 % ± 20 %) (Fig. 3B). The hierarchical expression pattern reflects the complex regulatory network in osteogenesis. BMP2′s positive expression (50 %) indicates its role as a key promoter of osteogenic differentiation, while the moderate negative expression of KDR (-20 %) suggests its involvement in coupling angiogenesis with osteogenesis. The negative expression of FGF10 (-50 %) might indicate its reduced role in later stages of differentiation. The master transcription factor RUNX2′s negative expression (-250 %) suggests a tightly regulated role in maintaining osteoblast commitment. The strong negative expression of PTK2 (-650 %) indicates significant downregulation during specific stages of osteogenic differentiation, while the differential expression of SMURF1 (-550 %) and SMURF2 (-150 %) suggests their role in fine-tuning BMP signaling through negative regulation. These significant differences in expression levels (*p* < 0.01) highlight the distinct and complementary roles of these genes in orchestrating the osteogenic differentiation process.

#### Chondrogenic differentiation

The analysis of gene expression involved in chondrogenesis revealed a complex pattern among eight key genes. The one-way ANOVA showed marginally significant differences among the expression groups (F = 5.1907, *p* = 0.0629). BMP2 demonstrated the highest positive expression (50 % ± 5 %), followed by moderate positive expression of GDF6 (15 % ± 2 %) and ITGAX (10 % ± 2 %). GDF5 and GDF7 showed similar low positive expression levels (both 5 % ± 1 %). In contrast, ABCB1 exhibited the strongest negative expression (−20 % ± 2 %), while BMP4 and KAT2B showed slight negative expression (both −5 % ± 1 %). (Fig. 3C). The hierarchical expression pattern reflects the regulatory network in chondrogenesis. BMP2′s strong positive expression (50 %) indicates its role as a key promoter of chondrogenic differentiation. The GDF family members (GDF5, GDF6, and GDF7) showed varying degrees of positive expression (5 %–15 %), suggesting their coordinated involvement in cartilage development. The moderate positive expression of ITGAX (10 %) implies its supportive role in chondrogenic processes. The negative expression of ABCB1 (−20 %) suggests its potential role as a negative regulator during chondrogenesis, while the slight negative expression of BMP4 and KAT2B (−5 %) indicates fine-tuning of the differentiation process. Although the differences in expression levels showed marginal statistical significance (*p* = 0.0629), the pattern reveals a complex interplay of positive and negative regulators in orchestrating chondrogenic differentiation.

### Elisa assay

**Figure 4:**
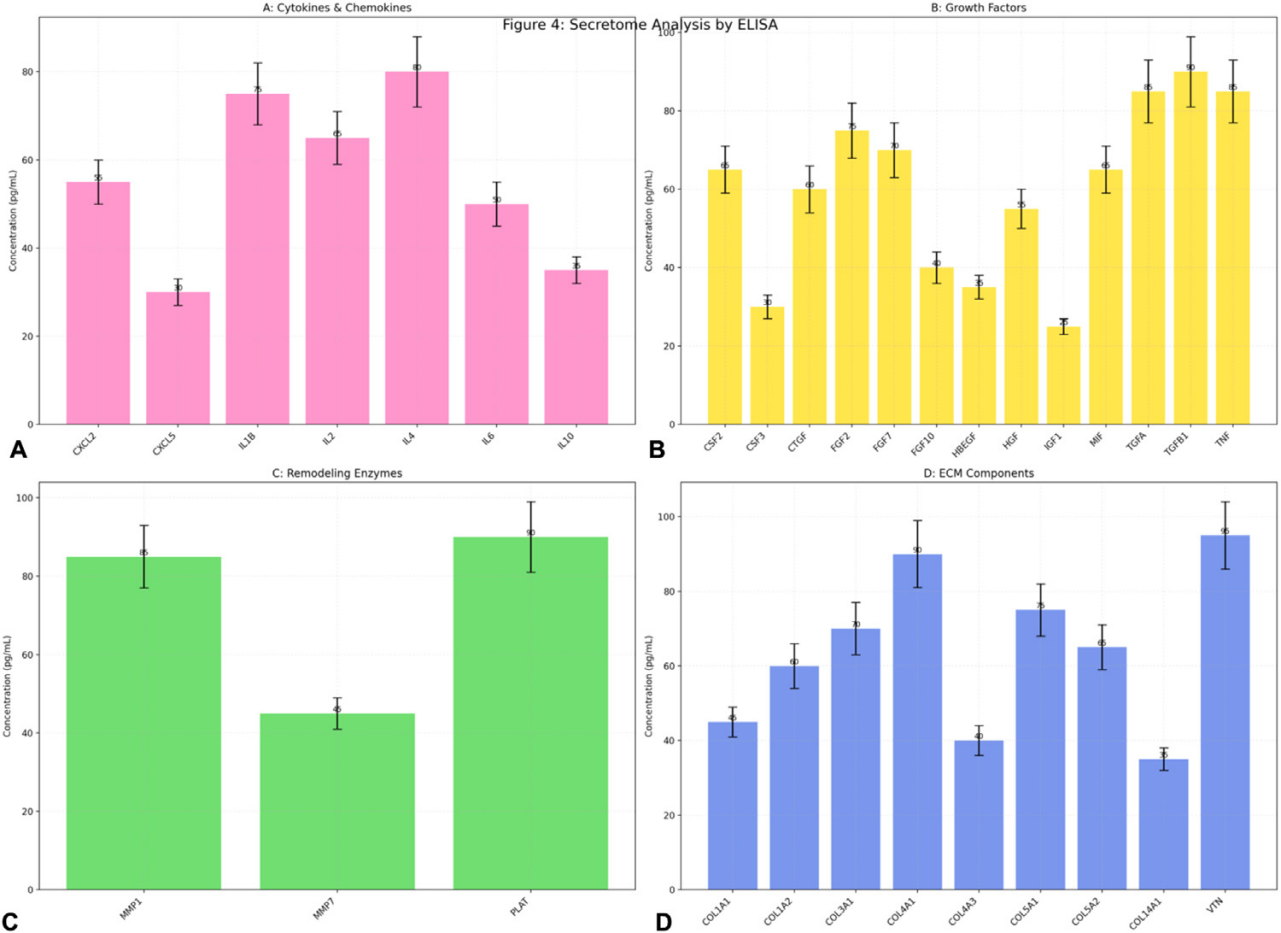
Comprehensive ELISA analysis of secretome components. ELISA quantification of secreted factors grouped into four major categories. A. Cytokines and chemokines profile showing the expression levels of inflammatory mediators (CXCL2, CXCL5) and interleukins (IL1B, IL2, IL4, IL6, IL10), highlighting the balance between pro- and anti-inflammatory signals. B. Growth factors analysis revealing the expression pattern of multiple growth-promoting factors, with notable high levels of TGFB1 (90 pg/mL) and TNF (85 pg/mL), indicating active tissue remodeling and regeneration processes. C. Remodeling enzymes measurement showing the relative concentrations of matrix metalloproteinases (MMP1, MMP7) and plasminogen activator (PLAT), demonstrating active ECM turnover. D. ECM components quantification displaying the expression levels of various collagen types (COL1A1, COL1A2, COL3A1, COL4A1, COL4A3, COL5A1, COL5A2, COL14A1) and vitronectin (VTN), indicating robust matrix production and organization. Data are presented as mean ± SD in pg/mL. All measurements were performed in triplicate.

The ELISA test results provide a comprehensive overview of the secretome profile, highlighting the interplay between cytokines, growth factors, remodeling enzymes, and ECM components (Figure 4). The cytokine profile (Figure 4A) reveals a balanced inflammatory response. IL1B and TNF exhibit significant pro-inflammatory activity, while IL4 and IL10 counterbalance with anti-inflammatory effects. CXCL2 and CXCL5 indicate moderate chemotactic activity, supporting immune cell recruitment. This balance suggests a controlled inflammatory environment conducive to tissue repair. The growth factor analysis (Figure 4B) shows TGFB1 and TNF as dominant players, driving both regenerative and inflammatory signaling. FGF2 and FGF7 indicate active fibroblast and epithelial proliferation, while IGF1′s low levels suggest limited involvement in this context. The robust growth factor signaling supports tissue regeneration and angiogenesis. The remodeling enzyme profile (Figure 4C) highlights PLAT and MMP1 as key contributors to ECM degradation and remodeling. MMP7 plays a secondary role, reflecting a tightly regulated ECM turnover. This activity is essential for maintaining tissue integrity and facilitating cellular migration. The ECM component analysis (Figure 4D) underscores the importance of VTN and COL4A1 in adhesion and basement membrane formation. COL1A2 and COL3A1 suggest robust collagen deposition, while COL14A1′s low levels indicate limited involvement in specialized ECM functions. This profile reflects active ECM remodeling and scaffold formation. The secretome profile reflects a dynamic and balanced microenvironment characterized by Controlled inflammation, Active ECM remodeling and Robust growth factor signaling This environment is ideal for tissue repair and regeneration, with a well-orchestrated interplay between pro-inflammatory and anti-inflammatory mediators, ECM turnover, and growth factor activity. The findings provide valuable insights into the molecular mechanisms underlying tissue remodeling and regeneration. The statistical analysis performed through ANOVA reveals that when comparing the concentration levels of different molecular categories, there is no statistically significant difference between these groups (*p* > 0.05, specifically *p* = 0.6582). This statistical finding suggests that while individual molecules within each category show varying concentrations, the overall mean expression levels across these different molecular categories maintain a relative balance. This balanced expression pattern indicates a well-coordinated molecular environment where different categories of signaling molecules, growth factors, and structural components are present in proportionate amounts, potentially supporting optimal tissue regeneration conditions.

### Clinical evaluation

The clinical study encompassed 171 patients who underwent lipofilling procedures utilizing the Body-jet system at our hospitals. A subset of these patients underwent multiple procedures during the study period, resulting in a total of 206 interventions.

Regarding the parameter of age, the mean patient age at the time of lipofilling was 52.77 years, with a recorded age range spanning from 36 to 73 years. (Supplemental Figure 5A). The mean recorded Body Mass Index (BMI) was 22.89. Specifically, 169 procedures were conducted on patients with a normal BMI range (18.5–24.9), 27 on overweight individuals (BMI 25–29.9), and 10 on obese patients (BMI >30). No procedures were performed on underweight patients. (Supplemental Figure 5B-D).

**Figure 5:**
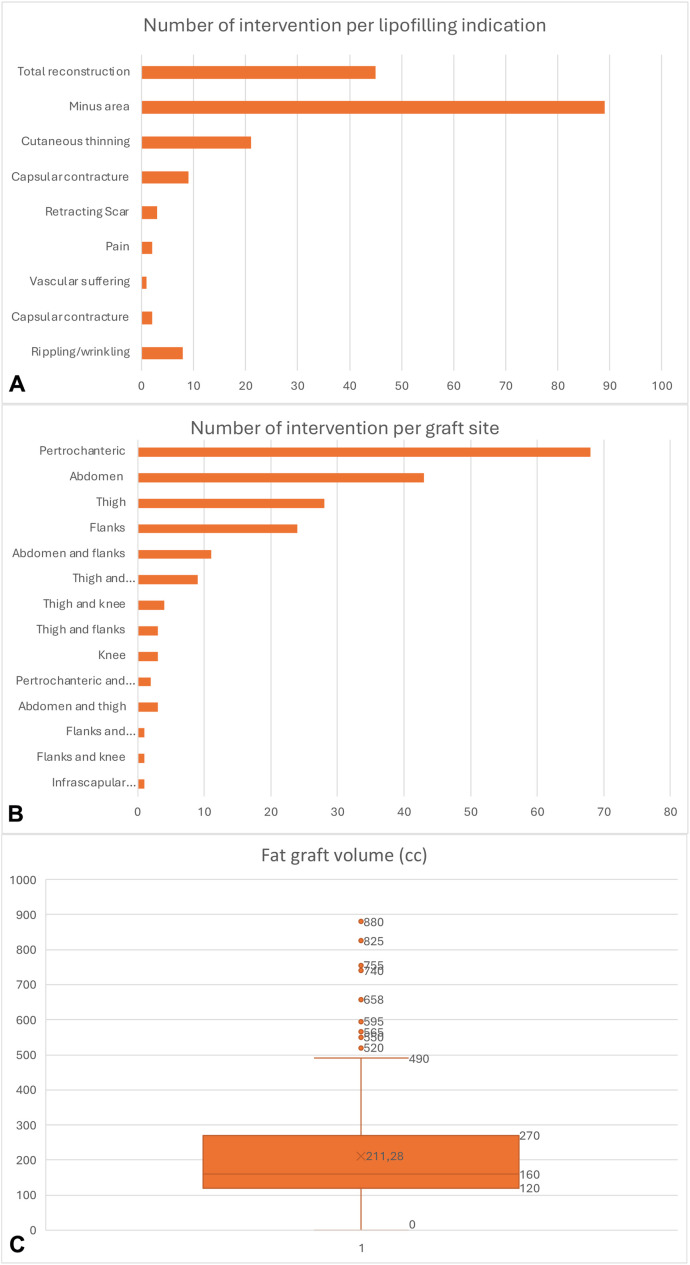
Detailed analysis of lipofilling procedures in the in vivo cohort. A. Number of interventions per clinical indication for lipofilling, including total breast reconstruction (n = 45), correction of volume deficits (n = 89), capsular contracture (n = 9), cutaneous thinning (n = 21), retracting scars (n = 3), pain (n = 2), skin vascular compromise (n = 2), combined capsular contracture and wrinkling (n = 2), and isolated wrinkling/rippling (n = 8); B. Number of procedures by selected fat harvesting site, including abdomen, peritrochanteric area, flanks, inner knee, and thighs; in some cases, two donor areas were used; C. Fat graft volume per patient (mean: 172 mL; range: 30–600 mL per procedure).

The cohort of patients had previously undergone five distinct primary surgical procedures: 17 patients had a quadrantectomy, 71 underwent radical mastectomy, 59 underwent nipple-sparing mastectomy, 30 received skin-sparing mastectomy, and 2 underwent skin-reducing mastectomy. Following the primary surgical intervention, 17 patients underwent autologous breast reconstruction. The indications for lipofilling included total breast reconstruction (45 patients), correction of volume deficits (89 patients), capsular contracture (9 patients), cutaneous thinning (21 patients), retracting scars (3 patients), pain (2 patients), vascular compromise of the skin (2), combined capsular contracture and wrinkling (2 patients), and wrinkling/rippling (8 patients). (Figure 5A). The fat harvesting sites included the abdomen, peritrochanteric region, flanks, inner knee surface, and thighs. In some procedures, fat was harvested from two of these areas (Figure 5B). Regarding fat grafting volume, an average of 172 mL of fat was harvested and infiltrated per procedure, with values ranging from 30 to 600 mL (Figure 5C). Regarding adjuvant therapies, 59 patients had undergone radiotherapy, 83 had received chemotherapy, and 114 had been treated with anti-hormonal therapy.

The comorbidities observed among patients included hypothyroidism (23 cases), depression (13), arterial hypertension (15), hypercholesterolemia (10), prior malignancy at an unrelated site (6), vasculopathy (6), cardiopathy (5), obstructive bronchopathy (4), gastroesophageal reflux disease (5), thrombophilia (3), arthritis (3), hyperthyroidism (2), hyperhomocysteinemia (2), ulcerative colitis (2), anemia (1), favism (1), and diabetes mellitus (1). Postoperative complications included a case of acute abdomen due to localized peritonitis from ileal perforation and hemoperitoneum (1), nodular formations at the donor site (1), and nodular formations at the recipient site (3). Additionally, breast carcinoma recurrence was reported in 8 patients. Data on postoperative pain 24 hours after the lipofilling procedure were retrieved from 170 medical records. Pain intensity was assessed using the Numeric Rating Scale (NRS), which ranges from 0 (no pain) to 10 (maximum pain). The mean NRS score reported was 3.17, with a standard deviation of 2.25.

**Figure 6:**
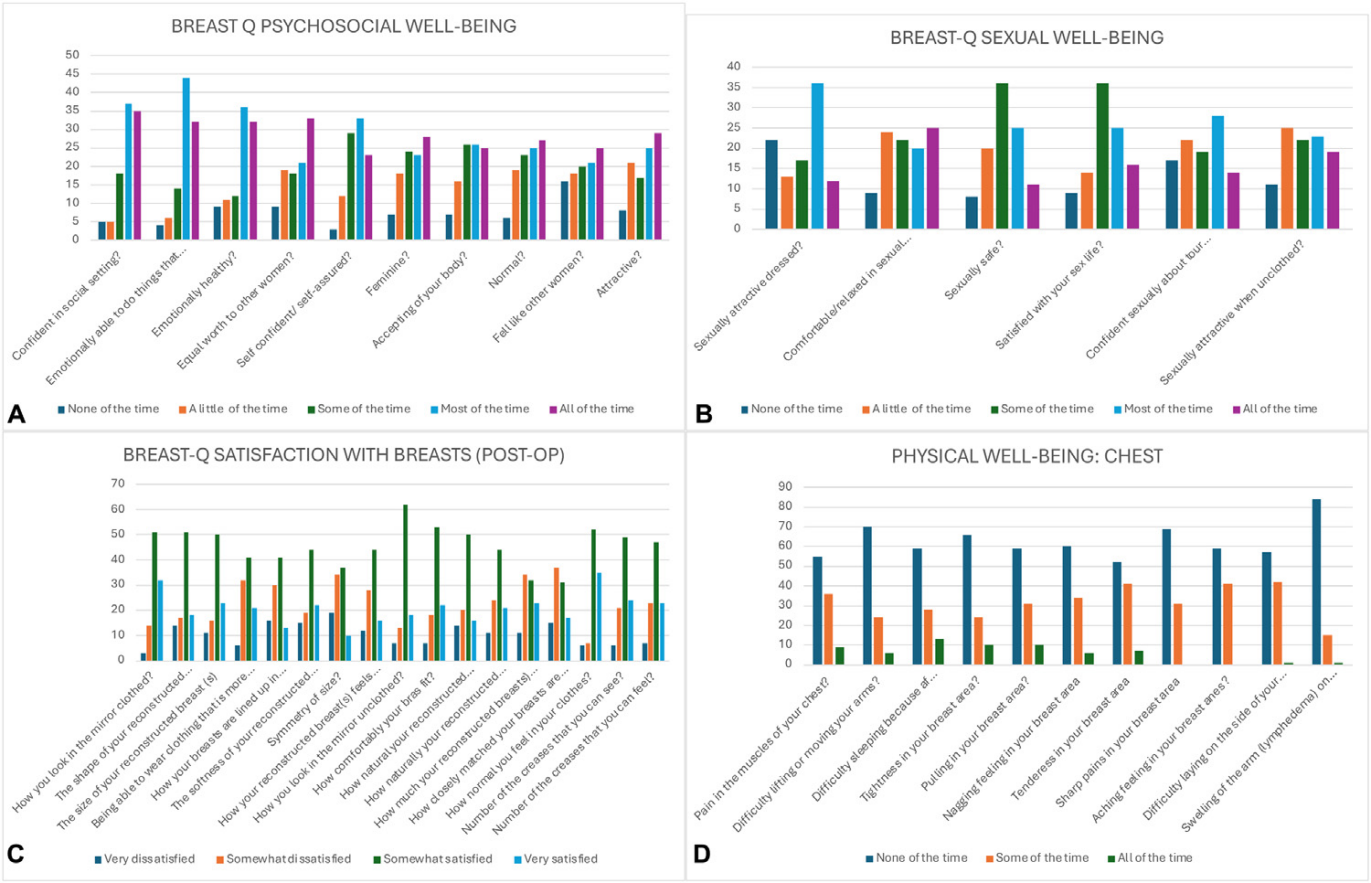
BREAST-Q questionnaire results evaluating quality of life and patient satisfaction after lipofilling. A. Psychosocial well-being; B. Sexual well-being; C. Postoperative satisfaction with breasts; D. Physical well-being of the chest. Scores reflect patients' subjective perceptions following the lipofilling procedure. Statistical analysis (Pearson’s correlation, *p* < 0.05) revealed a significant association between patient BMI and the volume of fat harvested for grafting (*p* < 0.001).

To evaluate patient satisfaction, all 171 individuals included in the study were contacted via telephone and invited to complete the BREAST-Q questionnaire. The survey was distributed electronically through Google Forms. A total of 100 patients responded within the specified timeframe, yielding a response rate of 58.5 % (Figure 6A-D). Statistical analysis, performed using Pearson’s correlation with a significance threshold of *p* < 0.05, revealed a statistically significant association between patient BMI and the volume of fat harvested for infiltration (*p* < 0.001).

Conversely, no significant correlation was observed between BMI and pain levels at 24 hours post-procedure (*p* = 0,2173). Likewise, the fat harvesting site demonstrated no statistically significant association with either the volume of fat obtained for infiltration (*p* = 0.3068) or postoperative pain at 24 hours (*p* = 0.6773). Furthermore, no significant correlation was identified between the volume of harvested fat and pain levels at 24 hours post-procedure (*p* = 0,2794) (Figure 6A-D).

## Discussion

In our experience, autologous fat grafting facilitates soft tissue remodeling through the transfer of adipose tissue harvested via liposuction to areas exhibiting volume deficiency. While technically straightforward, the procedure may induce transient postoperative complications at the donor site, including edema, ecchymosis, infection, and pain. Edema, a physiological response to cannula-induced trauma, is predominantly linked to impaired lymphatic drainage and excessive capillary filtration. Typically, it peaks around the fourth postoperative day and gradually resolves in the following weeks. Similarly, ecchymosis results from mechanical disruption of the superficial venous plexus, usually manifesting between days three and ten, and resolving spontaneously within two weeks. Pain following liposuction tends to be most intense during the first 24–72 hours, although it is usually well controlled with analgesics and decreases to mild levels over a period of two to three weeks.

These sequelae may limit patient willingness to undergo multiple lipofilling sessions during breast reconstruction. Nevertheless, autologous fat grafting remains the only viable option for patients with soft tissue deficits, capsular contracture, skin thinning, or wrinkling/rippling. For this reason, minimizing trauma during liposuction is essential in order to reduce postoperative discomfort and improve patient adherence to treatment plans. Moreover, the need for reliable alternatives to prosthetic-based reconstruction is particularly evident in patients undergoing post-mastectomy radiotherapy, where high complication rates often necessitate unplanned conversion to autologous techniques, as reported by Sedaghat et al. in a six-year clinical review.^18^ The Body-Jet liposuction system offers a solution to this challenge by employing a gentle, fan-shaped water jet that dislodges and simultaneously aspirates adipose tissue while preserving surrounding connective structures, nerves, and vessels. Previous studies have suggested that this method promotes a safer and less invasive procedure, with potentially superior aesthetic outcomes and reduced complications compared to traditional liposuction techniques.^14^^,^^19^^,^^21^ According to previous findings, reduced postoperative pain associated with WAL may contribute to improved patient compliance during staged procedures.^20^^,^^21^

As a body contouring technique, water-assisted liposuction (WAL) with the Body-Jet system was applied to 41 patients presenting with mild-to-moderate adiposity, with promising results and minimal complications.^19^ Despite requiring substantial tumescent volumes and yielding a nearly equal infiltration-to-aspiration ratio (1.1:1.0), the technique led to minimal blood loss and no adverse events necessitating intervention. Lidocaine dosages remained within safe thresholds (10.5 mg/kg for small volumes; 20.0 mg/kg for moderate volumes). Trypan blue exclusion testing revealed high adipocyte viability: approximately 90 % of cells remained viable 1 h after extraction and 10 % persisted up to 8 hours. Among 23 patients treated with fat grafting, early outcomes were promising, although further research is warranted to assess long-term efficacy and graft survival.^20^

Man and colleagues^21^ demonstrated that the pre-infiltration volume of tumescent solution was 20–30 % lower than in manual liposuction. Patient satisfaction exceeded 94 %, both in terms of procedural experience and final outcomes. Furthermore, the same authors^21^ reported a significant reduction in intraoperative swelling when using WAL compared to manual liposuction. The technique also enabled effective treatment of small fat deposits, responding to a growing demand for refined contouring. Importantly, pain reduction during and after WAL procedures has been documented in prior studies, suggesting improved tolerability and enabling faster return to daily activities.^18^, ^19^, ^20^, ^21^ Additional studies by Hoppe and colleagues^14^ confirmed these findings: with Body-Jet use, complication rates such as liponecrotic pseudocysts, infections, and granulomas were remarkably low, while satisfaction remained high (96 %), with 68 % of patients reporting favorable aesthetic results and natural breast softness and contour. This method also supports synchronous procedures such as nipple reconstruction or contralateral mastoplasty. In certain cases, temporary expanders were utilized to maintain or augment the skin envelope. Clinical evidence showed that 4 to 6 sessions, over 21 months, were often sufficient, with a mean operative time of 50 min and most patients discharged the same day. A surgical learning curve was observed, associated with reductions in operative time and complications.

Another advantage of Body-Jet liposuction is minimal bleeding, producing a blood-free lipoaspirate. Despite dilution by infiltrating fluid, the Lipocollector device facilitates substantial removal of the liquid phase. However, residual fluid likely remains within the adipose phase and is quickly reabsorbed post-injection, partially accounting for the reduced residual volume.^8^

Our retrospective analysis included 171 patients undergoing 206 procedures. The primary goal was to evaluate postoperative pain at 24 hours using the NRS scale and to identify correlations with BMI, donor site, or volume of harvested fat. Additionally, we analyzed the relationship between BMI and fat yield, and between donor site and tissue volume, alongside patient satisfaction one-year post-procedure using the BREAST-Q. These findings align with recent reports using BREAST-Q to assess patient-reported outcomes in breast surgery, such as the study by Graham et al.,^22^ which confirmed high satisfaction levels and improvements in psychosocial and sexual well-being following therapeutic mammaplasty and contralateral reshaping procedures.

A significant correlation was observed between BMI and fat volume (*p* < 0.001), confirming greater adipose yield in overweight patients. Pain scores at 24 hours were low (mean NRS = 3.17). These findings align with Araco and colleagues,^20^ who reported a mean VAS score of 2 at 24 hours post-WAL. No significant correlation emerged between postoperative pain and either BMI or donor site, nor between donor site and fat volume retrieved. The mean grafted fat volume (∼172 mL) was consistent with prior studies, confirming that this technique is comparable to traditional methods in terms of fat retrieval efficiency.

Patient satisfaction was high: 70 % expressed satisfaction with the shape, volume, and softness of reconstructed breasts. These observations are consistent with early findings by Rigotti and colleagues, who demonstrated the oncological safety and regenerative efficacy of autologous fat grafting in breast cancer patients, particularly in previously irradiated tissues.^23^ Notably, many reported improved self-confidence, body image, and femininity, with no cases of persistent breast discomfort.

In breast surgery, Body-Jet liposuction demonstrates significant potential, for example, in the surgical correction of tuberous breast deformity through fat grafting.^24^ In ten patients treated with percutaneous fasciotomies and fat transfer, BREAST-Q surveys showed significant improvements in satisfaction (0 to 75, *p* < 0.01), psychological well-being (20 to 70, *p* < 0.01), and sexual well-being (18.5 to 58, *p* = 0.02), while physical well-being remained stable. With a median satisfaction score of 86, this technique, based on our clinical results and those in the scientific literature,^24^^,^^25^ may offer an alternative to breast implants in selected cases, despite requiring multiple sessions. Furthermore, our results support the growing role of Body-Jet liposuction as a valid alternative for the correction of tuberous breasts in appropriately selected patients.^24^^,^^25^

The BEAULI method—Body-Jet-based fat harvesting—was validated in a study of 254 women (300 procedures), offering moderate augmentation, improved contour, and simultaneous body sculpting.^26^ Satisfaction rates were high (74.5 % rated outcomes as good/very good). While long-term data are still needed, this suggests a safe and natural approach to breast enhancement. For patients with severe capsular contracture (Baker III–IV), the Body-Jet method has proven to be an effective and minimally invasive alternative for patients with severe capsular contracture (Baker III-IV) following breast augmentation or reconstruction with implants. In a study of 64 patients (124 breasts), implant removal combined with autologous fat grafting achieved high patient satisfaction, yielding natural breast contours without complications such as oily cysts or infections. MRI assessments and clinical follow-ups confirmed stable outcomes, with an average fat graft volume of 260 mL and a procedure duration of approximately 70 min. This technique offers a promising solution for recurrent capsular contracture, providing a simpler, safer alternative to complex microvascular tissue transfers.^26^

Promising results have also emerged in regenerative medicine: in systemic sclerosis-related hand complications, Body-Jet–derived stromal vascular fraction (SVF) injected into necrotic zones prevented further amputations within three weeks without adverse events. Similarly, in vitro findings from our study showed rapid cell proliferation (tripling in 24 hours, doubling time ≈15 hours), indicating excellent preservation of adipose-derived mesenchymal stem cells (ASCs) and confirming their therapeutic potential.^6^

Bony and collegaues^8^ found that cells isolated via Body-Jet and manual methods were similar in viability and marker profiles, though manual liposuction yielded more cells per gram of fat. Our in vitro analysis demonstrated the remarkable proliferative capacity of cells derived from lipoaspirate processed using Body-Jet technology. The cells exhibited an exponential growth pattern, tripling in population within 24 hours, with a doubling time of approximately 15 hours and a consistent growth rate of 0.083 units/h. These findings indicate a highly viable and proliferative cell population, characteristic of adipose-derived mesenchymal stem cells. The observed rapid growth kinetics underscores the preservation of cell viability during the lipoaspiration process, highlighting the exceptional quality of the biological sample and confirms its significant potential for therapeutic applications. Similarly, the study by Palencar et al.^27^ found no statistically significant differences in the number, viability, or functional capacity of mesenchymal stem cells when comparing two different cell harvesting techniques.

However, ASCs from Body-Jet and LipiVage200–5 showed comparable CFU, metabolism, and multipotency, with Body-Jet yielding slightly faster proliferation and adipogenic potential.^28^ Yin and collegaues^7^ highlighted enhanced viability, reduced damage, and improved CD34+/CD45− ratios in WAL samples—factors linked to angiogenesis and graft survival. Water-jet-assisted liposuction yielded lipoaspirates with superior viability and a higher proportion of CD34/CD45 cells compared to conventional liposuction. Post-grafting, water-jet-assisted lipoaspirates exhibited enhanced weight retention, reduced apoptosis, and increased angiogenesis. These findings indicate that water-jet force positively influences the survival and regenerative potential of grafted fat, improving overall outcomes in liposuction procedures.^29^ Recent comparative studies have further highlighted how the technical approach to liposuction and graft processing significantly influences graft quality and retention. Hivernaud and colleagues compared four lipotransfer systems—manual, power-assisted, and water-assisted liposuction—followed by decantation, centrifugation, or filtration. Although all protocols yielded histologically viable grafts with minimal oil formation, the combination of manual aspiration with soft centrifugation and washing steps (Microfill®/Macrofill®) demonstrated superior tissue volume retention both in vitro and in vivo, outperforming Body-Jet (WAL) and PAL systems. These findings underscore the importance of processing techniques in optimizing graft integration and longevity.^30^ Similarly, Hartas and colleagues assessed adipocyte viability across dry suction-assisted liposuction (SAL), hyper-tumescent PAL, and WAL (Body-Jet). WAL yielded a significantly higher proportion of viable cells compared to hyper-tumescent PAL (*p* < 0.001), despite the latter achieving the highest overall cell count (*p* = 0.013). Dry SAL performed the worst in terms of viability (*p* = 0.011). These data suggest a trade-off between cell yield and quality, with WAL offering the most favorable balance for viable grafting.^31^

Our in vitro analyses provided compelling insights into the biological quality and regenerative potential of adipose tissue harvested via the Body-Jet system. Histological and molecular investigations, including ELISA and RT-qPCR, revealed a highly supportive microenvironment for tissue regeneration. Notably, we observed elevated expression of *WNT3A*, a key regulator of self-renewal and stemness, alongside robust expression of canonical stemness markers. Hierarchical clustering analysis of gene expression profiles identified three distinct expression clusters: a high-expression group centered on *WNT3A*, a medium-expression cluster comprising *INS* and *TERT*, and a low-expression group including *LIF, FGF2, POU5F1*, and *SOX2*. This stratification suggests the presence of metabolically active, partially committed progenitor cells, potentially transitioning toward specific mesenchymal lineages. Functional assays confirmed the multipotency of the harvested cells. Adipogenic, osteogenic, and chondrogenic differentiation was validated through the upregulation of lineage-specific markers such as *PPARG, BMP2*, and *GDFs*. This trilineage differentiation ability substantiates the mesenchymal identity and therapeutic potential of the isolated cell populations. Moreover, secretome profiling via ELISA indicated a well-orchestrated molecular landscape, characterized by balanced levels of cytokines, growth factors, and extracellular matrix components. No significant statistical differences were observed across molecular categories, suggesting a tightly regulated expression pattern that may foster efficient graft integration, angiogenesis, and remodeling post-transplantation. Although our study lacked a comparator arm, the cellular characteristics observed mirror those reported for high-viability grafts obtained via traditional methods, suggesting comparable or potentially superior biological behavior.^8^^,^^27^^,^^29^ Taken together, these findings support the hypothesis that water-assisted liposuction preserves a highly functional and viable stromal vascular fraction, suitable for applications in regenerative medicine.

In summary, our clinical and laboratory findings underscore the advantages of the Body-Jet system in adipose tissue harvesting, fat grafting, and regenerative medicine. This technique appears to provide a biologically high-quality graft product, reduced postoperative morbidity, and high patient satisfaction, as supported by our in vitro analyses, clinical evaluation in comparison with previous literature. Though transient side effects such as edema and bruising persist, the overall findings supports the growing use of WAL with Body-Jet in routine fat grafting and reconstructive procedures, in line with published clinical evidence. However, this study is not without limitations. Its retrospective nature, single-center design, and lack of randomization may introduce selection bias. Although our study lacked a direct control group, the clinical and laboratory outcomes observed with Body-Jet WAL appear consistent with results previously reported using conventional liposuction techniques. Future controlled studies will be necessary to directly validate these comparisons. Furthermore, the absence of a long-term follow-up limits our ability to evaluate graft retention and delayed complications. While in vitro findings suggest a high regenerative potential of the harvested cells, these results require further validation in standardized clinical trials to confirm their translational relevance.

## Conclusion

In conclusion, autologous fat grafting using the Body-Jet water-assisted liposuction system appears to be a promising approach for soft tissue reconstruction and body contouring. Based on our clinical experience and in vitro analyses, the procedure is associated with high adipocyte viability, low levels of postoperative pain, and rapid recovery, resulting in satisfactory aesthetic and reconstructive outcomes with a low incidence of complications. Supporting this, our in vitro findings confirmed the high viability and rapid proliferation of adipose-derived mesenchymal stem cells (ASCs), reinforcing the biological quality of the harvested tissue and its potential therapeutic applications. Nevertheless, transient effects such as edema and bruising remain present, and the retrospective, single-center nature of our study—combined with a lack of long-term follow-up—limits the generalizability of the results. Although our observations are consistent with existing literature on WAL techniques, direct comparisons were not performed. Future prospective studies with control groups, larger sample sizes, and extended follow-up periods are warranted to evaluate long-term graft retention, efficacy, and safety. Additionally, ongoing research should focus on refining harvesting and processing protocols to optimize both volumetric outcomes and regenerative capacity. Particular attention should be directed toward understanding the molecular mechanisms underpinning graft integration and ASC-driven regeneration. The integration of adjunct technologies—such as intraoperative imaging, 3D volumetric planning, and enhanced enrichment systems—may further improve procedural accuracy and outcome predictability. Finally, the favorable biological profile of Body-Jet–derived lipoaspirates suggests potential applicability beyond aesthetic surgery, including complex reconstructions, treatment of soft tissue pathologies, and regenerative approaches in autoimmune or ischemic diseases.

## Funding

The authors received no financial support for the research, authorship, and/or publication of this article.

## Informed consent

According to the policies of our institution, retrospective analyses of anonymized patient data do not require formal IRB approval. Nevertheless, the study adhered to all internal data protection protocols, and patient data were fully anonymized before analysis in compliance with applicable regulations.

## Institutional review board statement

Not applicable**.** This study was completed in accordance with the Declaration of Helsinki, as revised in 2013.

## Data availability

Data are contained within the article.

## Declaration of generative AI in scientific writing

No use.

## Author contributions

Conceptualization, F.D.F. and N.Z.; methodology, F.D.F; and N.Z.; validation, M.R., P.P., B.Z.; formal analysis, D.Q., G.M., A.D.P and M.P.C..; investigation, E.R.C.; resources, F.D.F., B.Z. and N.Z.; data curation, D.Q., G.M., A.D.P. and M.P.C; writing—original draft preparation, F.D.F and N.Z.; writing—review and editing, F.D.F.; visualization, P.P., B.Z. and M.R.; supervision, M.R., B.Z. and P.P.; project administration, M.R. All authors have read and agreed to the published version of the manuscript.

## Declaration of competing interest

The authors declare no potential conflicts of interest with respect to the research, authorship, and/or publication of this article.

## Appendix Supplementary materials

**SUPPLEMENTAL FIGURE 1:** Key components of the Body-Jet system used in water-assisted liposuction. A. Schematic illustration of the Lipocollector device. A primary sieve with 2.0 mm openings retains coarse connective tissue and larger fat clusters, which are less suitable for grafting. A secondary fine mesh filter (250 μm) isolates viable adipocytes from excess fluid; B. Dual-function cannula for concurrent irrigation and aspiration. Adipocytes are gently detached by the water stream, rather than being disrupted by mechanical force. C. Lipocollector® unit after liposuction, showing the harvested fat ready for grafting. Image, image 1

**SUPPLEMENTAL FIGURE 2:** A. Growth curve analysis showing experimental data points (blue dots) and fitted exponential growth model (red line, R² = 1.0000). The exponential fit demonstrates optimal cell proliferation with a population doubling time of 15.14 hours. Text inset shows key growth parameters; B. Instantaneous growth rate analysis over the culture period (green line). The shaded area represents the standard deviation of growth rates, while the red dashed line indicates the mean growth rate (0.0833 units/h). This analysis reveals consistent proliferation dynamics throughout the culture period. Scale: X-axis represents time in hours; Y-axis shows relative growth (panel A) and growth rate in units/h (panel B). Image, image 2

**SUPPLEMENTAL FIGURE 3:** A. Hierarchical Clustering of Stemness Markers. The dendrogram illustrates the hierarchical clustering of stemness markers based on their expression levels. Markers are grouped into three distinct clusters: High Expression Cluster: WNT3A (90 %); Medium Expression Cluster: INS (40 %) and TERT (30 %) and Low Expression Cluster: LIF (15 %), FGF2, POU5F1, and SOX2 (5 %). The Euclidean distance metric was used to calculate the dissimilarity between markers, and the Ward linkage method was applied to form the clusters. This analysis highlights the dominant role of WNT3A in stemness regulation and the hierarchical organization of other markers; B.Tukey's HSD post-hoc analysis of MSC-specific marker expression levels, illustrating statistically significant differences between high, moderate, and low expression groups. The hierarchical stratification underscores the differential regulation of molecular pathways in MSC characteristics. Image, image 3

**SUPPLEMENTAL FIGURE 4:** Demographic characterization of the in vivo lipofilling cohort. A. Age distribution of patients at the time of lipofilling (mean age 52.77 years; range 36–73 years); B. Distribution of Body Mass Index (BMI) among patients undergoing lipofilling with normal BMI (18.5–24.9, n = 169 procedures); C. Distribution of Body Mass Index (BMI) among patients undergoing lipofilling in overweight patients (BMI 25–29.9, n = 27); D. Distribution of Body Mass Index (BMI) among patients undergoing lipofilling in obese patients (BMI >30, n = 10). No procedures were performed on underweight patients. Image, image 4

**SUPPLEMENTAL FIGURE 5.** Correlation analysis between BMI, fat graft volume, and postoperative pain (NRS scale) 24 hours after the lipofilling procedure. A. Statistically significant correlation between BMI and harvested fat volume (*p* < 0.001). B. No significant correlation between BMI and pain scores at 24 hours post-procedure (NRS scale, *p* = 0.2173). C. No significant correlation between harvested fat volume and pain levels at 24 hours post-procedure (*p* = 0.2794). Additionally, the fat harvesting site showed no statistically significant association with either harvested fat volume (*p* = 0.3068) or postoperative pain levels (*p* = 0.6773). Image, image 5

## References

[1] Goncalves R., Mota B.S., Sobreira-Lima B. (2022). The oncological safety of autologous fat grafting: a systematic review and meta-analysis. BMC Cancer.

[2] Darrach H., Kraenzlin F., Khavanin N., Chopra K., Sacks JM. (2019). The role of fat grafting in prepectoral breast reconstruction. Gland Surg.

[3] Auclair E., Gianfermi M. (2021). Evaluation of a new adipose tissue processing method for breast and buttock fat grafting procedures. Eur J Plast Surg.

[4] Karina K., Biben J.A., Ekaputri K. (2025). Revisiting fat graft harvesting and processing technique to optimize its regenerative potential. Plast Reconstr Surg Glob Open.

[5] Hanson S.E., Kapur S.K., Hwang R.F., Dryden MS. (2021). Autologous fat grafting in breast reconstruction: implications for follow-up and surveillance. Gland Surg.

[6] Purpura V., Bondioli E., Melandri D., Parodi P.C., Valenti L., Riccio M. (2016). The collection of adipose derived stem cells using water-jet assisted lipoplasty for their use in plastic and reconstructive surgery: a preliminary study. Front Cell Dev Biol.

[7] Yin S., Luan J., Fu S., Wang Q., Zhuang Q. (2015). Does water-jet force make a difference in fat grafting? In vitro and in vivo evidence of improved lipoaspirate viability and fat graft survival. Plast Reconstr Surg.

[8] Bony C., Cren M., Domergue S., Toupet K., Jorgensen C., Noël D. (2016). Adipose mesenchymal stem cells isolated after manual or water-jet-assisted liposuction display similar properties. Front Immunol.

[9] Shukla L., Yuan Y., Shayan R., Greening D.W., Karnezis T. (2020). Fat therapeutics: the clinical capacity of adipose-derived stem cells and exosomes for human disease and tissue regeneration. Front Pharmacol.

[10] Hutchings G., Janowicz K., Moncrieff L. (2020). The proliferation and differentiation of adipose-derived stem cells in neovascularization and angiogenesis. Int J Mol Sci.

[11] Chen X., Yan L., Guo Z. (2016). Adipose-derived mesenchymal stem cells promote the survival of fat grafts via crosstalk between the Nrf2 and TLR4 pathways. Cell Death Dis.

[12] O’Halloran N., Courtney D., Kerin M.J., Lowery AJ. (2017). Adipose-derived stem cells in novel approaches to breast reconstruction: their suitability for tissue engineering and oncological safety. Breast Cancer (Auckl).

[13] Tang H., He Y., Liang Z., Li J., Dong Z., Liao Y. (2022). The therapeutic effect of adipose-derived stem cells on soft tissue injury after radiotherapy and their value for breast reconstruction. Stem Cell Res Ther.

[14] Hoppe D.L., Ueberreiter K., Surlemont Y., Peltoniemi H., Stabile M., Kauhanen S. (2013). Breast reconstruction de novo by water-jet assisted autologous fat grafting–a retrospective study. Ger Med Sci.

[15] Lauvrud A.T., Gümüscü R., Wiberg R. (2021). Water jet-assisted lipoaspiration and Sepax cell separation system for the isolation of adipose stem cells with high adipogenic potential. J Plast Reconstr Aesthet Surg.

[16] Gentile P., Casella D., Palma E., Calabrese C. (2019). Engineered fat graft enhanced with adipose-derived stromal vascular fraction cells for regenerative medicine: clinical, histological and instrumental evaluation in breast reconstruction. J Clin Med.

[17] Wu Q., Chen S., Peng W., Chen D. (2023). Current perspectives on cell-assisted lipotransfer for breast cancer patients after radiotherapy. World J Surg Oncol.

[18] Sedaghat N., Mousina R., Liu B. (2023). Surgical outcomes of post-mastectomy radiotherapy following immediate prosthetic breast reconstruction: six-year experience. The Breast.

[19] Sasaki GH. (2011). Water-assisted liposuction for body contouring and lipoharvesting: safety and efficacy in 41 consecutive patients. Aesthet Surg J.

[20] Araco A., Gravante G., Araco F., Delogu D., Cervelli V. (2007). Comparison of power water–assisted and traditional liposuction: a prospective randomized trial of postoperative pain. Aesthetic Plast Surg.

[21] Man D., Meyer H. (2007). Water jet-assisted lipoplasty. Aesthet Surg J.

[22] Graham S., Ihren I., Meybodi F., Hsu J., French J., Elder E. (2023). Therapeutic mammaplasty with contralateral symmetrising reduction mammaplasty: oncologically safe with satisfied patients. The Breast.

[23] Rigotti G., Marchi A., Micciolo R., Baroni G. (2012). Autologous fat grafting in breast cancer patients. The Breast.

[24] Papadopoulos S., Colpaert S.D.M., Vidovic G. (2024). Correction of the tuberous breast with fat grafting and implant: techniques, evaluation with BREAST-Q, and preliminary results. Aesthetic Plast Surg.

[25] Papadopoulos S., Colpaert S.D.M., Goulis D.G. (2021). Treating anisomastia and tuberous breast with fat grafting: technique and evaluation of outcomes using BREAST-Q surveys. Aesthetic Plast Surg.

[26] Ueberreiter K., Tanzella U., Cromme F., Doll D., Krapohl BD. (2013). One stage rescue procedure after capsular contracture of breast implants with autologous fat grafts collected by water assisted liposuction (“BEAULI Method”). GMS Interdiscip Plast Reconstr Surg DGPW.

[27] Palencar D., Dragunova J., Hulin I., Koller J. (2019). Adipose derived mesenchymal stem cells harvesting. Bratisl Lek Listy.

[28] Taha S., Saller M.M., Haas E. (2020). Adipose-derived stem/progenitor cells from lipoaspirates: a comparison between the Lipivage200-5 liposuction system and the Body-Jet liposuction system. J Plast Reconstr Aesthet Surg.

[29] Muench DP. (2016). Breast augmentation by water-jet assisted autologous fat grafting: a report of 300 operations. Surg J (N Y).

[30] Hivernaud V., Lefourn B., Robard M., Guicheux J., Weiss P. (2017). Autologous fat grafting: a comparative study of four current commercial protocols. J Plast Reconstr Aesthet Surg.

[31] Harats M., Millet E., Jaeger M. (2016). Adipocytes viability after suction-assisted lipoplasty: does the technique matter?. Aesthetic Plast Surg.

