# Supplementary figures and images for: Stemness and clinical performance of water-jet technology: A translational study in breast reconstruction
Source: JPRAS Open. 2025 Oct 24;46:750–69. doi: 10.1016/j.jpra.2025.10.010 (PMC12670530; doi:10.1016/j.jpra.2025.10.010)

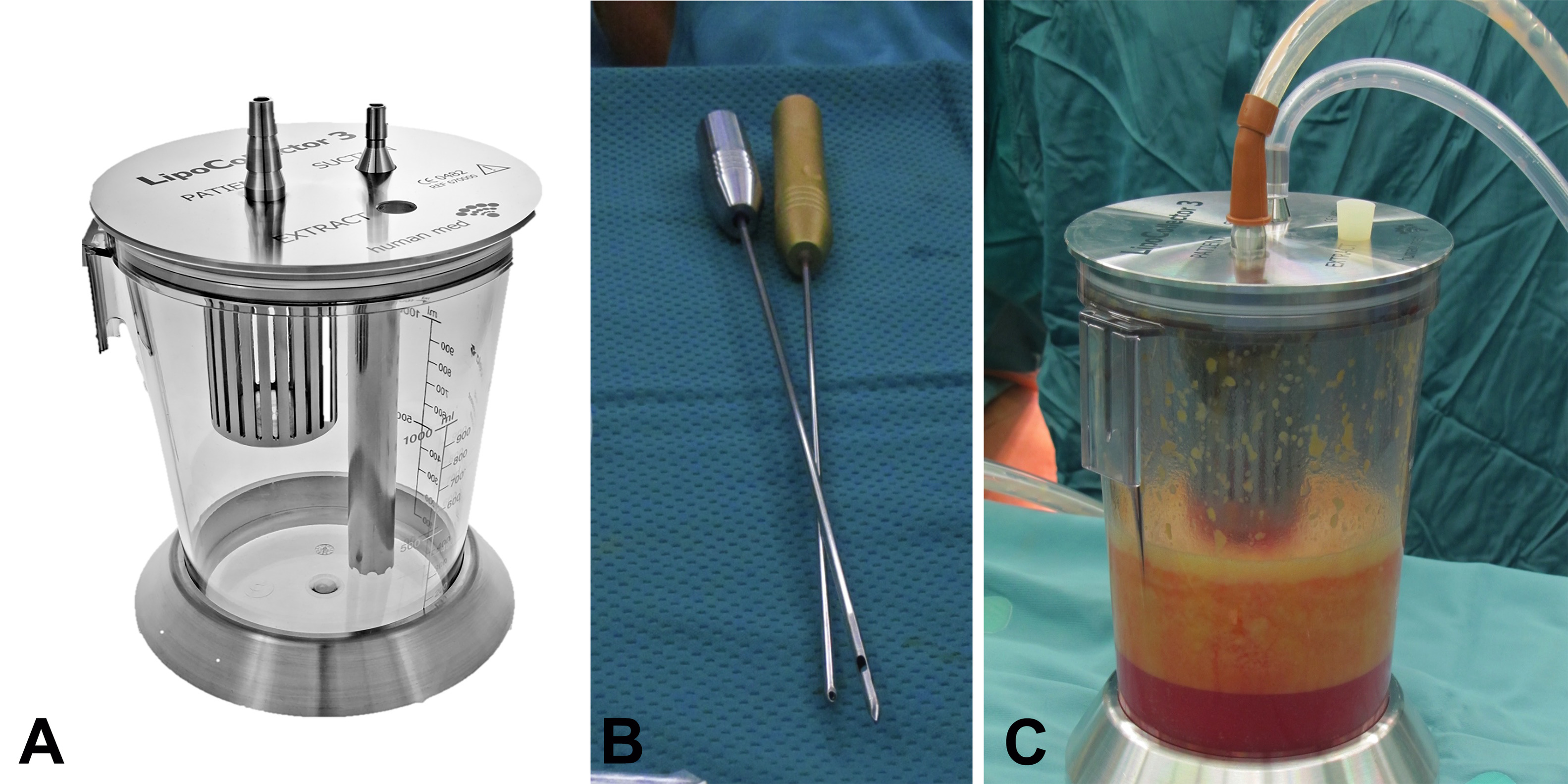

Supplement: Supplementary file 1 — SUPPLEMENTAL FIGURE 1: Key components of the Body-Jet system used in water-assisted liposuction. A. Schematic illustration of the Lipocollector device. A primary sieve with 2.0 mm openings retains coarse connective tissue and larger fat clusters, which are less suitable for grafting. A secondary fine mesh filter (250 μm) isolates viable adipocytes from excess fluid; B. Dual-function cannula for concurrent irrigation and aspiration. Adipocytes are gently detached by the water stream, rather than being disrupted by mechanical force. C. Lipocollector® unit after liposuction, showing the harvested fat ready for grafting. [file mmc1.jpg]

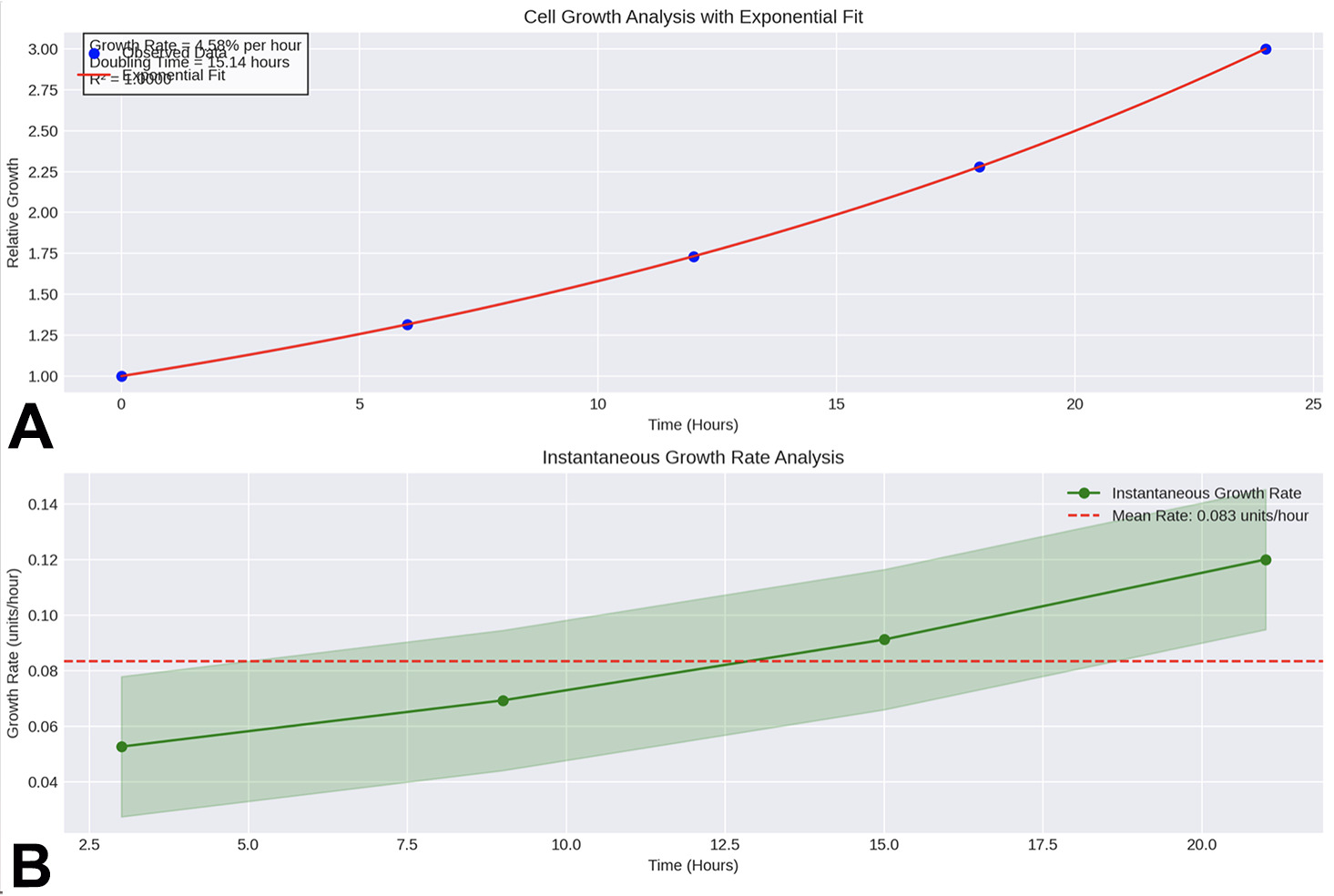

Supplement: Supplementary file 2 — SUPPLEMENTAL FIGURE 2: A. Growth curve analysis showing experimental data points (blue dots) and fitted exponential growth model (red line, R² = 1.0000). The exponential fit demonstrates optimal cell proliferation with a population doubling time of 15.14 hours. Text inset shows key growth parameters; B. Instantaneous growth rate analysis over the culture period (green line). The shaded area represents the standard deviation of growth rates, while the red dashed line indicates the mean growth rate (0.0833 units/h). This analysis reveals consistent proliferation dynamics throughout the culture period. Scale: X-axis represents time in hours; Y-axis shows relative growth (panel A) and growth rate in units/h (panel B). [file mmc2.jpg]

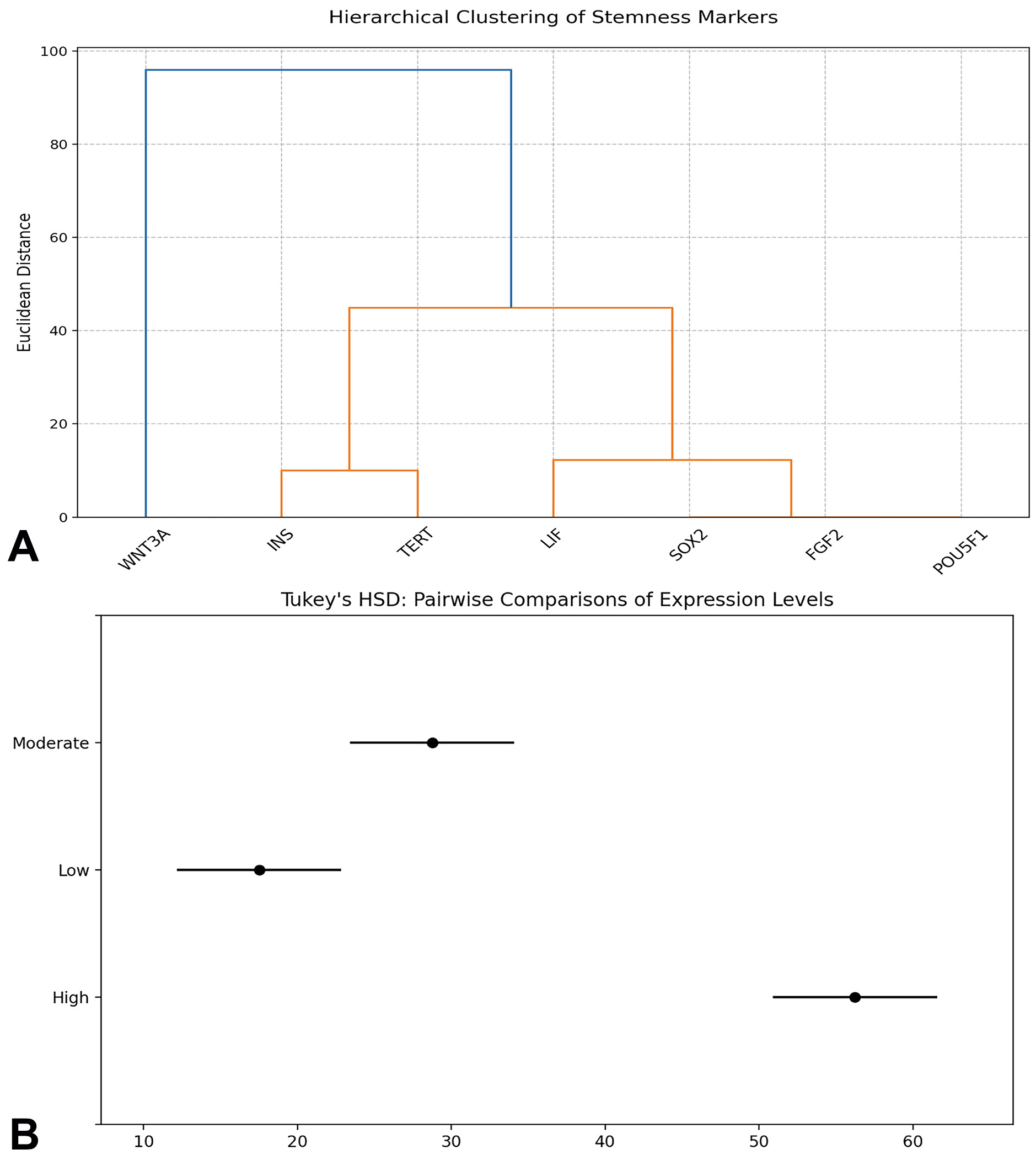

Supplement: Supplementary file 3 — SUPPLEMENTAL FIGURE 3: A. Hierarchical Clustering of Stemness Markers. The dendrogram illustrates the hierarchical clustering of stemness markers based on their expression levels. Markers are grouped into three distinct clusters: High Expression Cluster: WNT3A (90 %); Medium Expression Cluster: INS (40 %) and TERT (30 %) and Low Expression Cluster: LIF (15 %), FGF2, POU5F1, and SOX2 (5 %). The Euclidean distance metric was used to calculate the dissimilarity between markers, and the Ward linkage method was applied to form the clusters. This analysis highlights the dominant role of WNT3A in stemness regulation and the hierarchical organization of other markers; B.Tukey's HSD post-hoc analysis of MSC-specific marker expression levels, illustrating statistically significant differences between high, moderate, and low expression groups. The hierarchical stratification underscores the differential regulation of molecular pathways in MSC characteristics. [file mmc3.jpg]

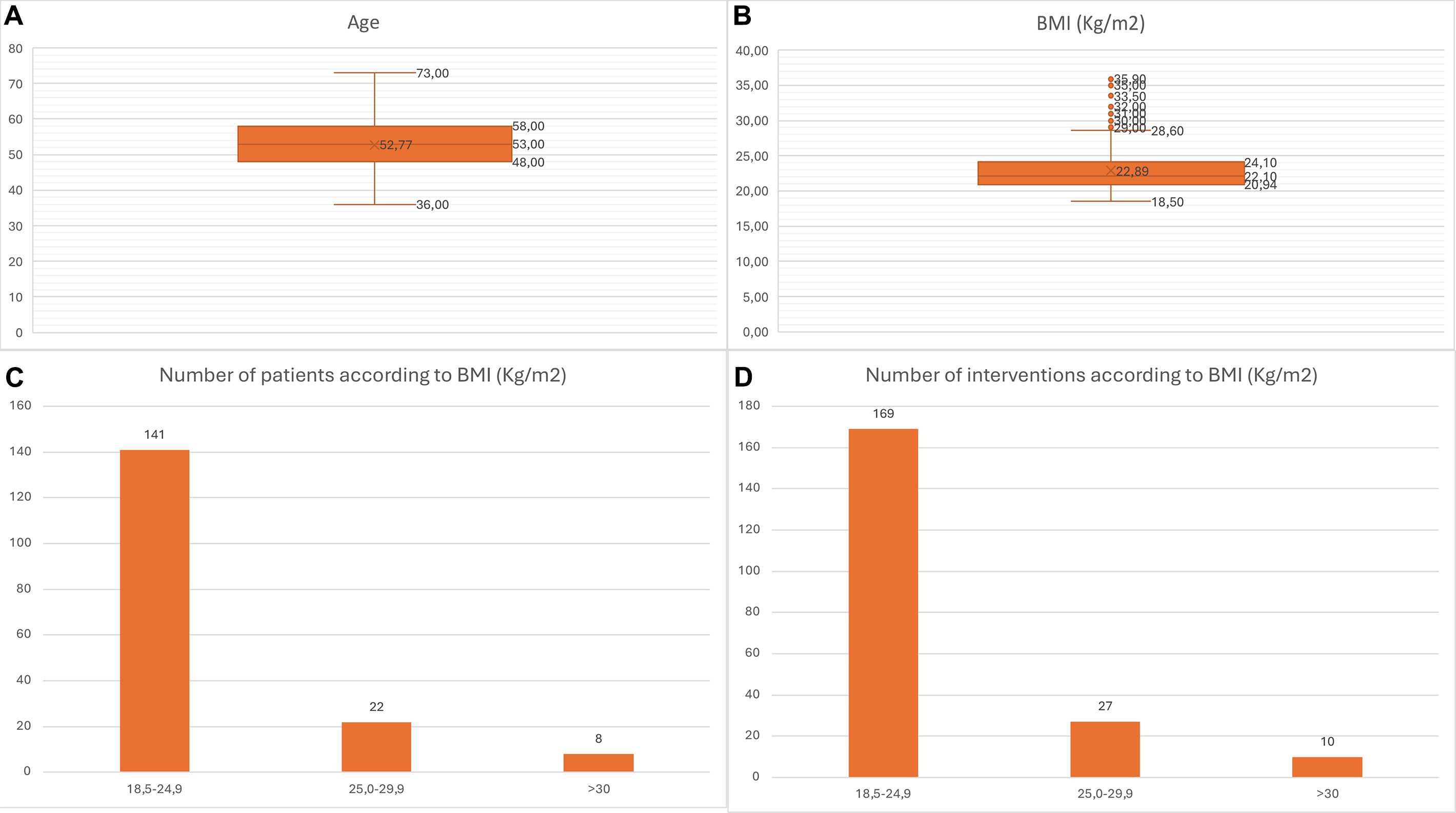

Supplement: Supplementary file 4 — SUPPLEMENTAL FIGURE 4: Demographic characterization of the in vivo lipofilling cohort. A. Age distribution of patients at the time of lipofilling (mean age 52.77 years; range 36–73 years); B. Distribution of Body Mass Index (BMI) among patients undergoing lipofilling with normal BMI (18.5–24.9, n = 169 procedures); C. Distribution of Body Mass Index (BMI) among patients undergoing lipofilling in overweight patients (BMI 25–29.9, n = 27); D. Distribution of Body Mass Index (BMI) among patients undergoing lipofilling in obese patients (BMI >30, n = 10). No procedures were performed on underweight patients. [file mmc4.jpg]

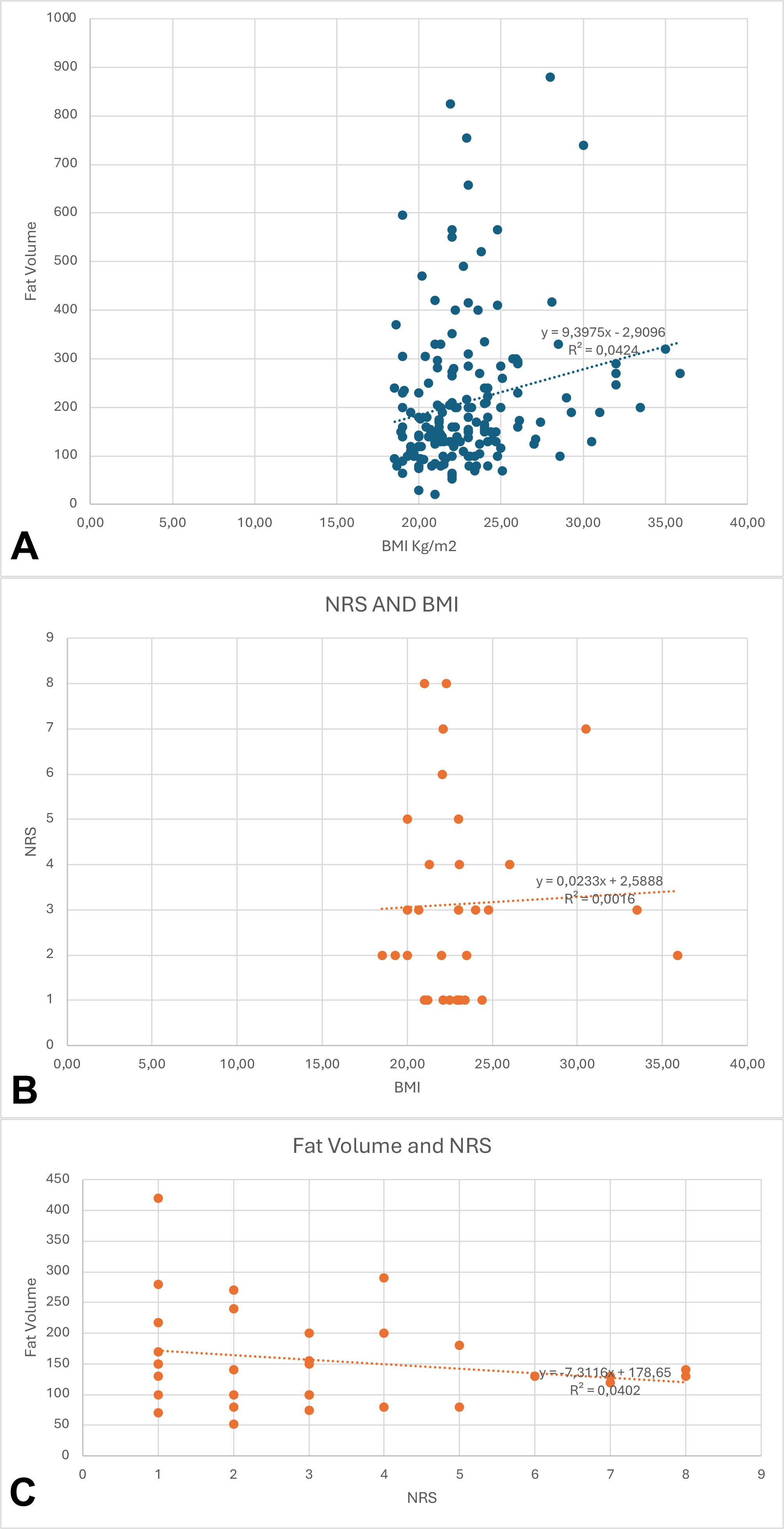

Supplement: Supplementary file 5 — SUPPLEMENTAL FIGURE 5. Correlation analysis between BMI, fat graft volume, and postoperative pain (NRS scale) 24 hours after the lipofilling procedure. A. Statistically significant correlation between BMI and harvested fat volume (p < 0.001). B. No significant correlation between BMI and pain scores at 24 hours post-procedure (NRS scale, p = 0.2173). C. No significant correlation between harvested fat volume and pain levels at 24 hours post-procedure (p = 0.2794). Additionally, the fat harvesting site showed no statistically significant association with either harvested fat volume (p = 0.3068) or postoperative pain levels (p = 0.6773). [file mmc5.jpg]
